# 1-[2-(2,4-Dichloro­benz­yloxy)-2-(2-fur­yl)eth­yl]-1*H*-1,2,4-triazole

**DOI:** 10.1107/S1600536809044018

**Published:** 2009-10-28

**Authors:** Özden Özel Güven, Hakan Tahtacı, Simon J. Coles, Tuncer Hökelek

**Affiliations:** a Department of Chemistry, Zonguldak Karaelmas University, 67100 Zonguldak, Turkey; b Department of Chemistry, Southampton University, SO17 1BJ Southampton, England; c Department of Physics, Hacettepe University, 06800 Beytepe, Ankara, Turkey

## Abstract

In the mol­ecule of the title compound, C_15_H_13_Cl_2_N_3_O_2_, the triazole ring is oriented at dihedral angles of 14.8 (2) and 81.5 (1)° to the furan and dichloro­benzene rings, respectively. The dihedral angle between the dichloro­benzene and furan rings is 86.3 (2)°. An intra­molecular C—H⋯O hydrogen bond results in the formation of a planar [maximum deviation 0.012 (2) Å] five-membered ring, which is oriented at a dihedral angle of 0.90 (7)° with respect to the dichloro­benzene ring. There is an inter­molecular C—H⋯π contact between the methyl­ene group and the dichloro­benzene ring.

## Related literature

For general background to the use of ether structures containing 1*H*-imidazole and 1*H*-1,2,4-triazole rings as anti­fungal agents, see: Caira *et al.* (2004 ▶); Godefroi *et al.* (1969 ▶); Özel Güven *et al.* (2007*a* ▶,*b* ▶); Paulvannan *et al.* (2001 ▶); Peeters *et al.* (1996 ▶); Wahbi *et al.* (1995 ▶). For related structures, see: Freer *et al.* (1986 ▶); Özel Güven *et al.* (2008*a* ▶,*b* ▶,*c* ▶,*d* ▶,*e* ▶,*f* ▶); Peeters *et al.* (1979 ▶).
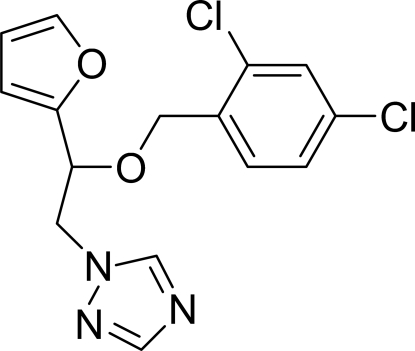


## Experimental

### 

#### Crystal data

C_15_H_13_Cl_2_N_3_O_2_
*M* *_r_* = 338.18
Monoclinic, 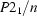

*a* = 10.6057 (2) Å
*b* = 13.3560 (3) Å
*c* = 11.1919 (2) Å
β = 101.170 (1)°
*V* = 1555.30 (5) Å^3^
*Z* = 4
Mo *K*α radiation
μ = 0.43 mm^−1^
*T* = 120 K
0.50 × 0.35 × 0.20 mm

#### Data collection

Bruker–Nonius Kappa CCD diffractometer
Absorption correction: multi-scan (*SADABS*; Sheldrick, 2007 ▶) *T*_min_ = 0.815, *T*_max_ = 0.920
6687 measured reflections
3547 independent reflections
2522 reflections with *I* > 2σ(*I*)
*R*_int_ = 0.025

#### Refinement

*R*[*F* ^2^ > 2σ(*F* ^2^)] = 0.052
*wR*(*F* ^2^) = 0.161
*S* = 1.06
3547 reflections
199 parameters
H-atom parameters constrained
Δρ_max_ = 0.77 e Å^−3^
Δρ_min_ = −0.33 e Å^−3^

### 

Data collection: *COLLECT* (Nonius, 1998 ▶); cell refinement: *DENZO* (Otwinowski & Minor, 1997 ▶) and *COLLECT*; data reduction: *DENZO* and *COLLECT*; program(s) used to solve structure: *SHELXS97* (Sheldrick, 2008 ▶); program(s) used to refine structure: *SHELXL97* (Sheldrick, 2008 ▶); molecular graphics: *ORTEP-3 for Windows* (Farrugia, 1997 ▶); software used to prepare material for publication: *WinGX* (Farrugia, 1999 ▶) and *PLATON* (Spek, 2009 ▶).

## Supplementary Material

Crystal structure: contains datablocks I, global. DOI: 10.1107/S1600536809044018/ci2951sup1.cif

Structure factors: contains datablocks I. DOI: 10.1107/S1600536809044018/ci2951Isup2.hkl

Additional supplementary materials:  crystallographic information; 3D view; checkCIF report

## Figures and Tables

**Table 1:** Hydrogen-bond geometry (Å, °)

*D*—H⋯*A*	*D*—H	H⋯*A*	*D*⋯*A*	*D*—H⋯*A*
C11—H11⋯O1	0.93	2.35	2.702 (3)	102
C9—H9*B*⋯*Cg*1^i^	0.97	2.90	3.775 (3)	151

Symmetry code: (i) 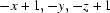. *Cg*1 is the centroid of the C10—C15 ring.

**Fig. 1.**
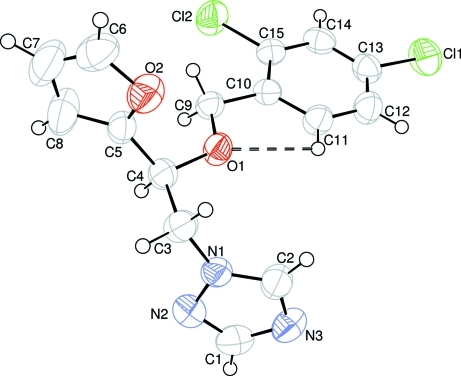
The molecular structure of the title compound with the atom-numbering scheme. Displacement ellipsoids are drawn at the 50% probability level. The dashed line indicates a hydrogen bond.

**Fig. 2.**
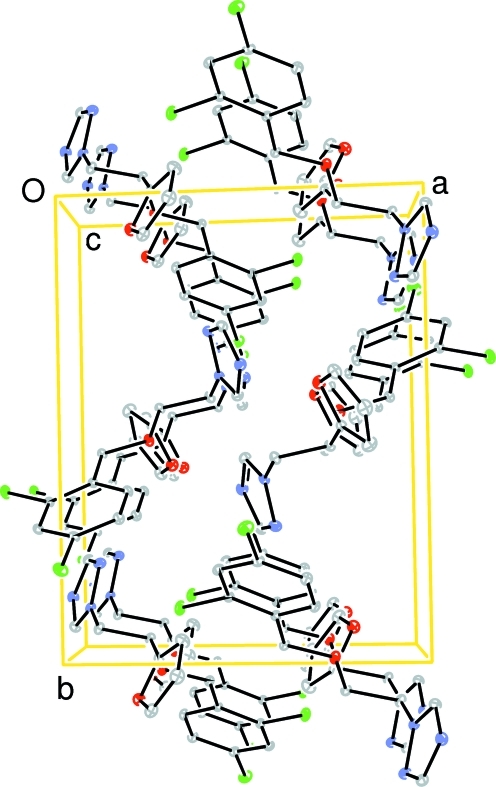
Part of a packing diagram. Hydrogen atoms have been omitted for clarity.

## supplementary crystallographic information

### Comment

In recent years, among antifungal agents, azole derivatives still have an important place in the class of systemic antifungal drugs. Some ether structures containing 1*H*-imidazole ring like micozanole, ecozanole and sulconazole have been synthesized and developed for clinical uses as antifungal agents (Godefroi *et al.*, 1969). The crystal structures of these ether derivatives like miconazole (Peeters *et al.*, 1979), econazole (Freer *et al.*, 1986) have been reported previously. Also, antifungal activity of aromatic ethers possessing 1*H*-1,2,4-triazole ring have been reported (Wahbi *et al.*, 1995). Itraconazole (Peeters *et al.*, 1996) and fluconazole (Caira *et al.*, 2004) are 1*H*-1,2,4-triazole ring containing azole derivatives. 1,2,4-Triazoles are biologically interesting molecules and their chemistry is receiving considerable attention due to antihypertensive, antifungal and antibacterial properties (Paulvannan *et al.*,  2001). Ether structures possessing 1*H*-benzimidazole ring have been reported to show antibacterial activity more than antifungal activity (Özel Güven *et al.*, 2007*a*,b). The crystal structures of 1*H*-benzimidazole ring containing ether derivatives (Özel Güven *et al.*, 2008*a*,b,c,d) and also,1*H*-1,2,4-triazole ring containing ether derivatives have been reported recently (Özel Güven *et al.*, 2008e,f). Now, we report herein the crystal structure of 2,4-dichloro- derivative of 1*H*-1,2,4-triazole and furyl rings containing ether structure.

In the molecule of the title compound (Fig. 1) the bond lengths and angles are generally within normal ranges. The planar triazole ring is oriented with respect to the furan and dichlorobenzene rings at dihedral angles of 14.8 (2)° and 81.5 (1)°, respectively. Atoms C3, C4 and C9 are -0.021 (2), 0.029 (2) and 0.034 (4) Å away from the planes of the triazole, furan and dichlorobenzene, respectively. So, they are nearly coplanar with the adjacent rings. The dichlorobenzene ring is oriented with respect to the furan ring at a dihedral angle of 86.3 (2)°. An intramolecular C—H···O hydrogen bond (Table 1) results in the formation of a planar five-membered ring (O1/H11/C9–C11), which is oriented with respect to dichlorobenzene ring at a dihedral angle of 0.90 (7)°. So, they are coplanar.

In the crystal, an intermolecular C—H···π interaction (Table 1) is observed between the methylene group and the dichlorobenzene ring. A view of the molecular packing in the crystal is shown in Fig.2.

### Experimental

The title compound was synthesized by the reaction of 1-(furan-2-yl)-2-(1*H*-1, 2,4-triazol-1-yl)ethanol (unpublished results) with NaH and appropriate benzyl halide. To a solution of alcohol (400 mg, 2.232 mmol) in DMF (4 ml) was added NaH (89 mg, 2.232 mmol) in small fractions. The appropriate benzyl halide (436 mg, 2.232 mmol) was added dropwise. The mixture was stirred at room temperature for 3 h, and excess hydride was decomposed with methyl alcohol (5 ml). After evaporation to dryness under reduced pressure, the crude residue was suspended with water and extracted with methylene chloride. The organic layer was dried over anhydrous sodium sulfate and then evaporated to dryness. The crude residue was purified by chromatography on a silica-gel column using chloroform as eluent. Crystals suitable for X-ray analysis were obtained by the recrystallization of the ether from 2-propanol (yield; 355 mg, 47%).

### Refinement

H atoms were positioned geometrically, with C-H = 0.93, 0.98 and 0.97 Å for aromatic, methine and methylene H, respectively, and constrained to ride on their parent atoms with *U*_iso_(H) = 1.2*U*_eq_(C).

### Crystal data

**Table:**

C_15_H_13_Cl_2_N_3_O_2_	*F*(000) = 696
*M**_r_* = 338.18	*D*_x_ = 1.444 Mg m^−^^3^
Monoclinic, *P*2_1_/*n*	Mo *K*α radiation, λ = 0.71073 Å
Hall symbol: -P 2yn	Cell parameters from 3274 reflections
*a* = 10.6057 (2) Å	θ = 2.9–27.5°
*b* = 13.3560 (3) Å	µ = 0.43 mm^−^^1^
*c* = 11.1919 (2) Å	*T* = 120 K
β = 101.170 (1)°	Plate, colourless
*V* = 1555.30 (5) Å^3^	0.50 × 0.35 × 0.20 mm
*Z* = 4	

### Data collection

**Table:**

Bruker–Nonius Kappa CCD diffractometer	3547 independent reflections
Radiation source: fine-focus sealed tube	2522 reflections with *I* > 2σ(*I*)
graphite	*R*_int_ = 0.025
φ and ω scans	θ_max_ = 27.5°, θ_min_ = 3.0°
Absorption correction: multi-scan (*SADABS*; Sheldrick, 2007)	*h* = −13→13
*T*_min_ = 0.815, *T*_max_ = 0.920	*k* = −17→17
6687 measured reflections	*l* = −14→14

### Refinement

**Table:**

Refinement on *F*^2^	Primary atom site location: structure-invariant direct methods
Least-squares matrix: full	Secondary atom site location: difference Fourier map
*R*[*F*^2^ > 2σ(*F*^2^)] = 0.052	Hydrogen site location: inferred from neighbouring sites
*wR*(*F*^2^) = 0.161	H-atom parameters constrained
*S* = 1.06	*w* = 1/[σ^2^(*F*_o_^2^) + (0.0852*P*)^2^ + 0.4221*P*] where *P* = (*F*_o_^2^ + 2*F*_c_^2^)/3
3547 reflections	(Δ/σ)_max_ = 0.001
199 parameters	Δρ_max_ = 0.77 e Å^−^^3^
0 restraints	Δρ_min_ = −0.32 e Å^−^^3^

### Special details

**Table:**

Geometry. All e.s.d.'s (except the e.s.d. in the dihedral angle between two l.s. planes) are estimated using the full covariance matrix. The cell e.s.d.'s are taken into account individually in the estimation of e.s.d.'s in distances, angles and torsion angles; correlations between e.s.d.'s in cell parameters are only used when they are defined by crystal symmetry. An approximate (isotropic) treatment of cell e.s.d.'s is used for estimating e.s.d.'s involving l.s. planes.
Refinement. Refinement of *F*^2^ against ALL reflections. The weighted *R*-factor *wR* and goodness of fit *S* are based on *F*^2^, conventional *R*-factors *R* are based on *F*, with *F* set to zero for negative *F*^2^. The threshold expression of *F*^2^ > σ(*F*^2^) is used only for calculating *R*-factors(gt) *etc*. and is not relevant to the choice of reflections for refinement. *R*-factors based on *F*^2^ are statistically about twice as large as those based on *F*, and *R*- factors based on ALL data will be even larger.

### Fractional atomic coordinates and isotropic or equivalent isotropic displacement parameters (Å^2^)

**Table:**

	*x*	*y*	*z*	*U*_iso_*/*U*_eq_	
Cl1	0.48306 (8)	0.29223 (6)	0.76970 (7)	0.0823 (3)	
Cl2	0.66147 (6)	0.12991 (6)	0.39547 (7)	0.0719 (3)	
O1	0.26246 (14)	0.02290 (13)	0.27553 (13)	0.0523 (4)	
O2	0.20740 (18)	0.07592 (15)	0.01684 (16)	0.0673 (5)	
N1	0.06121 (18)	−0.11249 (15)	0.27806 (16)	0.0498 (5)	
N2	0.0878 (2)	−0.20960 (17)	0.3093 (2)	0.0648 (6)	
N3	0.0001 (2)	−0.13162 (18)	0.45167 (19)	0.0644 (6)	
C1	0.0492 (3)	−0.2156 (2)	0.4135 (3)	0.0657 (7)	
H1	0.0556	−0.2746	0.4583	0.079*	
C2	0.0106 (3)	−0.0688 (2)	0.3648 (2)	0.0598 (6)	
H2	−0.0142	−0.0019	0.3637	0.072*	
C3	0.0914 (2)	−0.0710 (2)	0.16712 (19)	0.0550 (6)	
H3A	0.0425	−0.0100	0.1464	0.066*	
H3B	0.0658	−0.1183	0.1011	0.066*	
C4	0.2337 (2)	−0.04811 (18)	0.17976 (19)	0.0495 (5)	
H4	0.2831	−0.1093	0.2036	0.059*	
C5	0.2625 (2)	−0.01101 (19)	0.0610 (2)	0.0505 (5)	
C6	0.2450 (3)	0.0932 (3)	−0.0918 (2)	0.0760 (8)	
H6	0.2218	0.1486	−0.1416	0.091*	
C7	0.3176 (4)	0.0206 (3)	−0.1140 (3)	0.0926 (11)	
H7	0.3551	0.0147	−0.1823	0.111*	
C8	0.3303 (3)	−0.0490 (3)	−0.0155 (3)	0.0884 (10)	
H8	0.3765	−0.1086	−0.0071	0.106*	
C9	0.3934 (2)	0.05052 (18)	0.30581 (19)	0.0460 (5)	
H9A	0.4171	0.0885	0.2396	0.055*	
H9B	0.4468	−0.0089	0.3191	0.055*	
C10	0.4136 (2)	0.11323 (16)	0.41992 (18)	0.0430 (5)	
C11	0.3143 (2)	0.13351 (19)	0.4814 (2)	0.0528 (6)	
H11	0.2325	0.1087	0.4508	0.063*	
C12	0.3349 (2)	0.1896 (2)	0.5868 (2)	0.0590 (6)	
H12	0.2674	0.2025	0.6267	0.071*	
C13	0.4554 (2)	0.22648 (19)	0.6326 (2)	0.0547 (6)	
C14	0.5566 (2)	0.20958 (18)	0.5743 (2)	0.0532 (6)	
H14	0.6379	0.2351	0.6052	0.064*	
C15	0.5333 (2)	0.15318 (17)	0.4678 (2)	0.0470 (5)	

### Atomic displacement parameters (Å^2^)

**Table:**

	*U*^11^	*U*^22^	*U*^33^	*U*^12^	*U*^13^	*U*^23^
Cl1	0.1025 (6)	0.0819 (6)	0.0616 (4)	−0.0194 (4)	0.0135 (4)	−0.0290 (4)
Cl2	0.0461 (4)	0.0894 (6)	0.0834 (5)	−0.0112 (3)	0.0204 (3)	−0.0183 (4)
O1	0.0435 (8)	0.0694 (11)	0.0444 (8)	−0.0108 (7)	0.0093 (6)	−0.0156 (7)
O2	0.0722 (12)	0.0746 (13)	0.0579 (10)	0.0045 (10)	0.0191 (8)	0.0056 (9)
N1	0.0488 (10)	0.0542 (12)	0.0468 (10)	−0.0102 (9)	0.0101 (8)	−0.0052 (8)
N2	0.0735 (14)	0.0536 (13)	0.0701 (14)	−0.0059 (11)	0.0206 (11)	−0.0055 (10)
N3	0.0621 (13)	0.0746 (16)	0.0615 (12)	0.0017 (11)	0.0249 (10)	0.0094 (11)
C1	0.0627 (16)	0.0617 (17)	0.0743 (17)	−0.0078 (13)	0.0171 (13)	0.0120 (13)
C2	0.0644 (15)	0.0610 (16)	0.0577 (13)	0.0051 (12)	0.0213 (11)	0.0013 (12)
C3	0.0521 (13)	0.0703 (16)	0.0415 (11)	−0.0148 (11)	0.0069 (9)	−0.0046 (10)
C4	0.0505 (12)	0.0546 (14)	0.0431 (11)	−0.0075 (10)	0.0086 (9)	−0.0083 (10)
C5	0.0482 (12)	0.0583 (14)	0.0466 (11)	−0.0061 (11)	0.0133 (9)	−0.0116 (10)
C6	0.081 (2)	0.095 (2)	0.0525 (14)	−0.0173 (18)	0.0141 (13)	0.0096 (15)
C7	0.101 (2)	0.125 (3)	0.0621 (17)	−0.008 (2)	0.0413 (17)	−0.0092 (18)
C8	0.099 (2)	0.100 (3)	0.0766 (19)	0.0192 (19)	0.0428 (18)	−0.0125 (17)
C9	0.0430 (11)	0.0520 (13)	0.0437 (10)	−0.0073 (10)	0.0101 (8)	−0.0030 (9)
C10	0.0439 (11)	0.0428 (12)	0.0416 (10)	−0.0036 (9)	0.0067 (8)	0.0020 (8)
C11	0.0455 (12)	0.0633 (16)	0.0495 (12)	−0.0082 (10)	0.0094 (9)	−0.0073 (10)
C12	0.0594 (14)	0.0677 (16)	0.0518 (13)	−0.0041 (12)	0.0157 (11)	−0.0110 (11)
C13	0.0670 (15)	0.0499 (14)	0.0456 (12)	−0.0063 (11)	0.0072 (10)	−0.0063 (10)
C14	0.0548 (13)	0.0495 (14)	0.0508 (12)	−0.0109 (10)	−0.0009 (10)	0.0008 (10)
C15	0.0434 (11)	0.0479 (13)	0.0493 (11)	−0.0030 (9)	0.0083 (9)	0.0026 (9)

### Geometric parameters (Å, °)

**Table:**

Cl1—C13	1.742 (2)	C5—C8	1.321 (3)
Cl2—C15	1.740 (2)	C6—H6	0.93
O1—C4	1.419 (3)	C7—C6	1.293 (5)
O1—C9	1.413 (2)	C7—H7	0.93
O2—C5	1.350 (3)	C8—C7	1.428 (5)
O2—C6	1.370 (3)	C8—H8	0.93
N1—N2	1.358 (3)	C9—H9A	0.97
N1—C2	1.332 (3)	C9—H9B	0.97
N1—C3	1.451 (3)	C10—C15	1.386 (3)
N2—C1	1.311 (3)	C10—C11	1.391 (3)
N3—C1	1.341 (4)	C10—C9	1.508 (3)
N3—C2	1.305 (3)	C11—C12	1.379 (3)
C1—H1	0.93	C11—H11	0.93
C2—H2	0.93	C12—H12	0.93
C3—H3A	0.97	C13—C12	1.373 (3)
C3—H3B	0.97	C14—C13	1.378 (4)
C4—C3	1.519 (3)	C14—C15	1.391 (3)
C4—C5	1.504 (3)	C14—H14	0.93
C4—H4	0.98		
			
C9—O1—C4	114.40 (16)	C6—C7—C8	108.0 (3)
C5—O2—C6	106.9 (2)	C6—C7—H7	126.0
N2—N1—C3	121.0 (2)	C8—C7—H7	126.0
C2—N1—N2	108.9 (2)	C5—C8—C7	105.6 (3)
C2—N1—C3	130.1 (2)	C5—C8—H8	127.2
C1—N2—N1	101.6 (2)	C7—C8—H8	127.2
C2—N3—C1	101.9 (2)	O1—C9—C10	108.67 (17)
N2—C1—N3	116.1 (2)	O1—C9—H9A	110.0
N2—C1—H1	121.9	O1—C9—H9B	110.0
N3—C1—H1	121.9	C10—C9—H9A	110.0
N1—C2—H2	124.3	C10—C9—H9B	110.0
N3—C2—N1	111.4 (2)	H9A—C9—H9B	108.3
N3—C2—H2	124.3	C11—C10—C9	122.02 (19)
N1—C3—C4	112.17 (18)	C15—C10—C9	120.72 (19)
N1—C3—H3A	109.2	C15—C10—C11	117.3 (2)
N1—C3—H3B	109.2	C10—C11—H11	119.4
C4—C3—H3A	109.2	C12—C11—C10	121.3 (2)
C4—C3—H3B	109.2	C12—C11—H11	119.4
H3A—C3—H3B	107.9	C11—C12—H12	120.1
O1—C4—C5	113.35 (19)	C13—C12—C11	119.8 (2)
O1—C4—C3	105.63 (18)	C13—C12—H12	120.1
O1—C4—H4	109.0	C12—C13—Cl1	119.7 (2)
C3—C4—H4	109.0	C12—C13—C14	121.2 (2)
C5—C4—C3	110.63 (18)	C14—C13—Cl1	119.08 (19)
C5—C4—H4	109.0	C13—C14—C15	118.0 (2)
O2—C5—C4	117.3 (2)	C13—C14—H14	121.0
C8—C5—O2	110.1 (3)	C15—C14—H14	121.0
C8—C5—C4	132.5 (3)	C10—C15—Cl2	119.41 (17)
O2—C6—H6	125.3	C10—C15—C14	122.5 (2)
C7—C6—O2	109.3 (3)	C14—C15—Cl2	118.09 (18)
C7—C6—H6	125.3		
			
C9—O1—C4—C3	177.01 (19)	C3—C4—C5—C8	−113.9 (3)
C9—O1—C4—C5	−61.7 (3)	O2—C5—C8—C7	0.9 (4)
C4—O1—C9—C10	−171.48 (18)	C4—C5—C8—C7	178.2 (3)
C6—O2—C5—C4	−178.8 (2)	C8—C7—C6—O2	−0.2 (4)
C6—O2—C5—C8	−1.0 (3)	C5—C8—C7—C6	−0.4 (4)
C5—O2—C6—C7	0.8 (3)	C11—C10—C9—O1	2.1 (3)
C2—N1—N2—C1	0.4 (3)	C15—C10—C9—O1	−177.9 (2)
C3—N1—N2—C1	178.7 (2)	C9—C10—C11—C12	178.9 (2)
N2—N1—C2—N3	−0.9 (3)	C15—C10—C11—C12	−1.1 (4)
C3—N1—C2—N3	−179.0 (2)	C9—C10—C15—Cl2	−0.3 (3)
N2—N1—C3—C4	−77.0 (3)	C9—C10—C15—C14	−178.6 (2)
C2—N1—C3—C4	100.9 (3)	C11—C10—C15—Cl2	179.67 (18)
N1—N2—C1—N3	0.2 (3)	C11—C10—C15—C14	1.4 (3)
C2—N3—C1—N2	−0.7 (3)	C10—C11—C12—C13	0.0 (4)
C1—N3—C2—N1	1.0 (3)	Cl1—C13—C12—C11	−176.9 (2)
O1—C4—C3—N1	−60.3 (3)	C14—C13—C12—C11	0.9 (4)
C5—C4—C3—N1	176.7 (2)	C15—C14—C13—Cl1	177.24 (18)
O1—C4—C5—O2	−55.2 (3)	C15—C14—C13—C12	−0.6 (4)
O1—C4—C5—C8	127.7 (3)	C13—C14—C15—C10	−0.6 (4)
C3—C4—C5—O2	63.3 (3)	C13—C14—C15—Cl2	−178.91 (19)

### Hydrogen-bond geometry (Å, °)

**Table:**

*D*—H···*A*	*D*—H	H···*A*	*D*···*A*	*D*—H···*A*
C11—H11···O1	0.93	2.35	2.702 (3)	102
C9—H9B···Cg1^i^	0.97	2.90	3.775 (3)	151

Symmetry codes: (i) −*x*+1, −*y*, −*z*+1.
